# Adverse effects of Influenza A(H1N1)pdm09 virus infection on growth performance of Norwegian pigs - a longitudinal study at a boar testing station

**DOI:** 10.1186/s12917-014-0284-6

**Published:** 2014-12-04

**Authors:** Chiek Er, Bjørn Lium, Saraya Tavornpanich, Peer Ola Hofmo, Hilde Forberg, Anna Germundsson Hauge, Carl Andreas Grøntvedt, Tore Framstad, Edgar Brun

**Affiliations:** Norwegian Veterinary Institute, P.O. Box 750, 0106 Oslo, Norway; Norsvin (Norwegian Pig Breeders Association), P.O. Box 504, 2304 Hamar, Oslo Norway; Norwegian University of Life Sciences, Campus Adamstuen, Ullevålsveien 72, 0454 Oslo, Norway

**Keywords:** Growth performance, Daily growth, Feed conversion efficiency, Longitudinal study, Multi-level regression analysis, Random intercept model, Pigs, Feed intake, Influenza A(H1N1)pdm09 virus

## Abstract

**Background:**

Influenza A(H1N1)pdm09 virus infection in Norwegian pigs was largely subclinical. This study tested the hypothesis that the infection causes negligible impact on pigs’ growth performance in terms of feed conversion efficiency, daily feed intake, daily growth, age on reaching 100 kg bodyweight and overall feed intake. A sample of 1955 pigs originating from 43 breeding herds was classified into five infection status groups; seronegative pigs (n = 887); seropositive pigs (n = 874); pigs positive for virus at bodyweight between 33 kg and 60 kg (n = 123); pigs positive for virus at bodyweight between 61 kg and 80 kg (n = 34) and pigs positive for virus at bodyweight between 81 kg and 100 kg (n = 37). Each pig had daily recordings of feed intake and bodyweight from 33 kg to 100 kg. Marginal effects of the virus infection on the outcomes were estimated by multi-level linear regression, which accounted for known fixed effects (breed, birthdate, average daily feed intake and growth phase) and random effects (cluster effects of pig and herd).

**Results:**

The seropositive and virus positive pigs had decreased (P value<0.05) growth performance compared to seronegative pigs even though feed intake was not decreased. Reduced feed conversion efficiency led to lower average daily growth, additional feed requirement and longer time needed to reach the 100 kg bodyweight. The effects were more marked (P value<0.03) in pigs infected at a younger age and lasted a longer period. Despite increased feed intake observed, their growth rates were lower and they took more time to reach 100 kg bodyweight compared to the seronegative pigs.

**Conclusion:**

Our study rejected the null hypothesis that the virus infection had negligible adverse effects on growth performance of Norwegian pigs.

## Background

Respiratory diseases in pigs are serious concerns for pig producers worldwide because they cause substantial economic losses from increased mortality, reduced feed efficiency and growth rate, increased time to reach market weight, increased carcass condemnation at slaughter and costs of treatment and vaccination [1]. Among the many respiratory pathogens in pigs, swine influenza viruses (SIVs) are ubiquitous in intensive pig farming, and can be primary agents in causing respiratory disease [2-4]. In April 2009, a new influenza A virus, named influenza A(H1N1)pdm09 emerged in North- and South-America. It spread rapidly in humans and pigs and soon became endemic in pig populations worldwide including Norway [5-7]. Like other SIVs, this A(H1N1)pdm09 virus spreads easily between pigs and can cause acute respiratory disease [8-10] characterized by high fever, depression, loss of appetite, tachypnoea, abdominal breathing and coughing [7]. Uncomplicated SIV infections cause low mortality (usually less than 1%), but morbidity can reach 100% [7]. Respiratory disease caused by SIVs can be exacerbated by concurrent infections of other respiratory pathogens of which Porcine Reproductive and Respiratory Syndrome virus (PRRSV), *Mycoplasma hyopneumoniae, Pasteurella multocida,* and *Actinobacillus pleuropnuemoniae* are the most common. Such respiratory diseases are most frequently detected in 10- to 22-week-old pigs and are termed the porcine respiratory disease complex [1,11].

As compared to other pig populations in the world, Norwegian pigs have the favourable condition of being free from many respiratory pathogens like PRRSV, porcine respiratory coronavirus and *M. hyopneumoniae*, which are serious respiratory pathogens in almost all pig producing countries. Up until 2009, the Norwegian pig population had also been free from SIVs. However in the autumn of 2009, the Norwegian pig population experienced the first outbreak of influenza virus infection [12]. Pig farmers and farm workers infected with influenza A(H1N1)pdm09 virus transmitted the virus to the pigs [13,14]. Within a few months, one third of Norwegian pig herds were positive for antibodies against the virus [15]. Subsequent annual national surveillance from 2010 to 2012 revealed that 41-50% of pig herds were seropositive, indicating that the virus had become endemic in the Norwegian pig population [12]. Farmers of positive herds reported mild or absence of clinical signs in their pigs [16] in Norway and in other parts of the world [7].

Although there have been studies investigating the clinical signs, pathology and immunology related to SIVs, including influenza A(H1N1)pdm09 virus infection in pigs [2,9-11,17,18], there is little information available on the adverse effects of influenza virus infection on growth performance of pigs in the field. We therefore aimed by using a field study to investigate the adverse effects of influenza A(H1N1)pdm09 virus on pig production performance with longitudinal growth performance data from a pig testing station between 2009 and 2012. Given the mild clinical picture presented in previous studies from Norway [14-16], the present study tested the hypothesis that influenza A(H1N1)pdm09 virus has little or no impact on growth performances in infected grower pigs. The study also investigated whether the virus infection had different impacts on Landrace and Duroc breeds, on pigs infected at different ages, and the duration of the adverse effects if present.

## Methods

### Boar testing station

During our period of investigation between 2009 and 2012, the boar testing station in Norway [19] had a capacity of testing 1152 pigs in 16 separate rooms at the same time. Each room housed a cohort of 72 pigs grouped by breed (Landrace or Duroc) into six groups of 12 pigs and placed in pens of 14 m^2^ in size. The station received weekly, 72 growing pigs (11–12 weeks old with a mean bodyweight of 33 kg) from 46 breeding herds in Norway to monitor their growth performances until they reached a bodyweight of 100 kg. Electronic feeding stations in all pig pens used FIRE (Feed Intake Recording Equipment, Osbourne Ltd, UK) to record daily feed intake and daily weight gain of each pig individually. Pigs fed one at a time *ad libitum* from one electronic feed dispenser in each pen on conventional concentrate containing 161 g and 136 g digestible protein, 9.68 MJ, and 9.50 MJ net energy/kg before and after 50 kg live weight, respectively, with 1 month of mixing the two feeds to facilitate the feed change.

### Study sample

**Figure 1:**
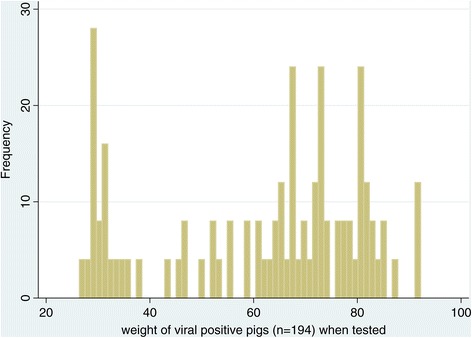
**Histogram of weight of virus positive pigs (n= 194) when they were tested positive for virus shedding.**

The study sample consisted of 1955 pigs (55% Landrace, 45% Duroc) from 43 breeding herds that were performance tested at the testing station between 2009 and 2012. All pigs were tested for antibodies against influenza A virus by cELISA before leaving the station. During a clinical outbreak of influenza at the boar station between 1^st^ April 2011 and 31^st^ July 2011, nasal swabs and blood samples were collected from a total of 375 of these pigs (three pigs per pen) to test for presence of virus and antibodies against the virus, respectively. The testing methods have been described previously [15]. Based on laboratory findings, the 1955 pigs were classified into five infection status groups (INFGP). The seronegative group (SERONEG) with 887 pigs was defined as pigs tested negative for antibodies against influenza A(H1N1)pdm09 virus at the end of their performance testing period. The seropositive group (SEROPOS) with 874 pigs was defined as pigs tested positive for antibodies against influenza A(H1N1)pdm09 virus but with unknown point of infection. Most (n = 859) of these pigs were tested at the end of the performance testing period when they were about 100 kg in weight thus ruling out maternal antibodies in cELISA test results. The virus positive group one (VIR1) with 123 pigs was defined as pigs tested positive for influenza A(H1N1)pdm09 virus by RT-PCR when their bodyweight was between 33 kg and 60 kg (growth phase one or GF1). Of these VIR1 pigs, twenty-two were in the upper weight range of between 51 kg and 60 kg when they were tested positive for the virus. The virus positive group two (VIR2), with 34 pigs, was defined as pigs tested positive for influenza A(H1N1)pdm09 virus by RT-PCR when their bodyweight was between 61 kg and 80 kg (growth phase two or GF2). The virus positive group three (VIR3), with 37 pigs was defined as pigs that tested positive for influenza A(H1N1)pdm09 virus by RT-PCR when their bodyweight was between 81 kg and 100 kg (growth phase three or GF3). Figure 1 is a histogram showing the distribution of the bodyweights of these 194 virus positive pigs when they were tested. These pigs were then classified into three groups to ensure that there were at least 30 pigs in each group.

During the period when the station experienced a clinical outbreak of influenza A(H1N1)09pdm virus, the staff intensified the clinical observation of the 2045 pigs that stayed at the station during this period. The source of the outbreak was not investigated as the infection has become widespread in the Norwegian pig population since its incursion into Norway in 2009 [15].

### Statistical analysis

Growth and feeding performances were represented by five outcomes; average daily growth (ADG, weight gain in kg/day), feed conversion efficiency (FCE, kg feed/kg weight gain), average daily feed intake (ADFI, feed intake in kg/day), age of pig when they reached 100 kg (Age100kg) bodyweight; and the overall feed intake (OFI) of the pigs to grow from a mean starting weight of 33 kg to 100 kg. Besides the infection status groups (INFGP; SERONEG, SEROPOS, VIR1, VIR2 and VIR3) as the main predictor of interest, important control predictors were birthdate (BD), breed (Br), ADFI, and GF. For each of the three outcomes of ADG, FCE and ADFI, every pig had three measures aggregated for the three stipulated growth phases. The SERONEG pigs were the reference group for comparison with the SEROPOS group and the three virus positive groups. The data was structured longitudinally on daily measures of growth and feed intake [19] and was hierarchical with three levels. The observations for each outcome were nested in the 1955 pigs, which in turn were nested in the 43 herds. To estimate the negative effects of the virus infection on growth performance, we used multi-level random-intercept regression models to control for other important predictors, while accounting for clustering or random effects at the pig and herd levels [20]. We used analysis of covariance to determine the statistical significance (P value<0.05 for level of inclusion) of predictors for each of the five outcomes. Predictors BD (proxy for improvement over time from 2009 to 2012) and ADFI were continuous variables while INFGP, Br, GF and two interaction terms of GF*INFGP and Br*INFGP for stratification were categorical variables. The INFGP*GF interaction term was included in the regression model to investigate the modifying effect of age of infection on the virus [21]. The INFGP*Br interaction term was tested for statistical significance to see if the impact of the virus infection was different between Landrace and Duroc. The two outcomes, Age100 kg and OFI, were single measures for each pig’s overall performance when they reached 100 kg bodyweight. The predictors for them were the same as for FCE and ADG, but without GF since the measures were aggregated over all the 3 growth phases. To select the best multi-level models for the five outcomes with the selected predictors, we used Akaike Information Criterion (AIC). We selected the model with the lowest AIC value. To determine the significance of additional predictors for each of the five models (one outcome per model), a difference of ±2 of the AIC value was regarded as non-significant and the most parsimonious model was then chosen [22].

### Multi-level random-intercept regression models

$$ {Y}_{\left[i,j,k\right]} = {\beta}_0+{\beta}_1{X}_{1\left[i,j,k\right]}+\dots .. + {\beta}_h{X}_{h\left[i,j,k\right]} + {u}_{\left[j,k\right]}+{v}_{\left[k\right]} + {\varepsilon}_{\left[i,j,k\right]}. $$

*Where:*

Y is one of the five outcomes in this study (ADG, FCE, ADFI, Age100kg, or OFI,). *Y*_*ijk*_ is the value of the response for *i*th (i = 1,2,3) observation for *j*th pig (n_*j*_*=* 1955) nested within the *k*th (n_*k*_*=* 43) herd.

*β* is a vector of coefficients for predictors and their interactions, and *X*_*[i,j,k]*_ is the vector of explanatory variables for the *i*th observation of the *j*th pig and *k*th herd.

*u*_*jk*_ is a vector of random intercepts unique to each pig in each herd, where *u*_*jk*_ ~ *N*(0, σ^2^_pig_), and *v*_*k*_ is a vector of random intercepts unique to each herd, where *v*_*k*_ ~ *N*(0, σ ^2^_herd_).

*ε*_ijk_ is the vector of error terms where *ε*_ijk_ ~ *N*(μ, σ^2^).

### Predictors

Apart from BD and ADFI that are continuous predictors, the following are categorical predictors:

- **INFGP:** SERONEG; SEROPOS; VIR1; VIR2; VIR3.
- **Br:** Landrace; Duroc
- **GF:** GF1 (33 kg to 60 kg); GF2 (61 kg to 80 kg); GF3 (81 kg to 100 kg).

Quantitative bias analysis by Episens [23] was used to estimate the magnitude of misclassification bias for adverse effects on FCE given that the cELISA test had a respective sensitivity and specificity of 93.7% and 99.1% (manufacturer’s data sheet). A small number of misclassification of the 887 seronegative pigs and 874 seropositive pigs may have been possible. This required dichotomizing the continuous outcome FCE into high and low with median FCE value of 2.54 for the seronegative pigs chosen for the dichotomy.

We used software SAS Enterprise Guide 4.3 (SAS Institute Inc., Cary, NC, USA) and STATA version 12.0 (StataCorp LP, College Station, TX, USA) for data handling and statistical analysis. We plotted the predicted marginal effects of the five infection status groups of pigs (INFGP) for each of our five outcomes based on our regression models by keeping the other covariates at the sample mean values.

### Ethics

This was a field study that involved pigs at a commercial pig testing station. No pigs were harmed during the collection of blood samples from the jugular vein or nasal swabs to ascertain their infection status.

## Results

### Statistical models

Based on AIC values and the parsimony principle, Table 1 shows the final multi-level models for the five outcomes. For outcomes ADG, FCE and ADFI, there were three levels (5,865 observations, 1955 pigs, and 43 herds) in the hierarchy. For the remaining two outcomes, Age100 kg and OFI, there were two levels (1955 observations and 43 herds) in the hierarchy. Only one interaction term of interest, between infection status and growth phase (INFGP*GF) was statistically significant indicating that the effect of the virus varied with the different growth phases. The second interaction of practical interest between infection status and breed (INFGP*Br), was found statistically insignificant during model selection indicating that that effects of the virus infection on Landrace and Duroc were similar or the power was insufficient to reject the null hypothesis.

**Table 1:** **Akaike information criterion (AIC) and the parsimony principle were used to select the best model for each outcome**

**Outcome**	**Predictors (fixed effects)**							**Random effects**		**AIC**	**ΔAIC**
	**Infection status group (INFGP)**	**Breed (Br)**	**Growth Phase (GF)**	**Birthdate**	**Ave daily feed intake (ADFI)**	Interaction term INFGP*GF	Interaction term INFGP*Br	**Pig**	**Herd**		
**ADG**	x	x	x	x	x	x	x	x	x	-7157.8	-1.7
	x	x	x	x	x	x	x		x	-7132.5	23.6
	x	x	x	x	x	x	x	x		-7147.8	8.3
→	**x**	**x**	**x**	**x**	**x**	**x**		**x**	**x**	**-7156.1**	**0**
	x	x	x	x	x		x	x	x	-7150	6.1
	x	x	x	x	x			x	x	-7149	7.1
**FCE**	x	x	x	x	x	x	x	x	x	-1889	3.7
	x	x	x	x	x	x	x		x	-1769	123.7
	x	x	x	x	x	x	x	x		-1878	14.7
→	**x**	**x**	**x**	**x**	**x**	**x**		**x**	**x**	**-1892.7**	**0**
	x	x	x	x	x		x	x	x	-1864	28.7
	x	x	x	x	x			x	x	-1867	25.7
**ADFI**	x	x	x	x	x	x	x	x	x	3299.8	2.8
	x	x	x	x	x	x	x		x	3564	267
	x	x	x	x	x	x	x	x		3307	10
→	**x**	**x**	**x**	**x**	**x**	**x**		**x**	**x**	**3297**	**0**
	x	x	x	x	x		x	x	x	3304	7
	x	x	x	x	x			x	x	3302	5
**Age 100 kg**	x	x		x	x		x		x	13111	5
	x	x		x	x		x			13205.5	99.5
→	**x**	**x**		**x**	**x**				**x**	**13106**	**0**
**OFI**	x	x		x	x		x		x	15281	3
	x	x		x	x		x			15362	84
→	**x**	**x**		**x**	**x**				**x**	**15278**	**0**

*Abbreviations*: *ADG* average daily growth in kg/day, *FCE* feed conversion efficiency in kg feed/kg weight gain, *ADFI* average daily feed intake in kg/day, *Age 100 kg* age of pig when they reached 100 kg, *OFI* overall feed intake in kg from 33 kg to 100 kg bodyweight.

Selected model is indicated by (→). Marking X indicates predictor was included in the model. ΔAIC = difference in AIC between the tested models and the selected model given in bold.

### ADG and FCE

**Figure 2:**
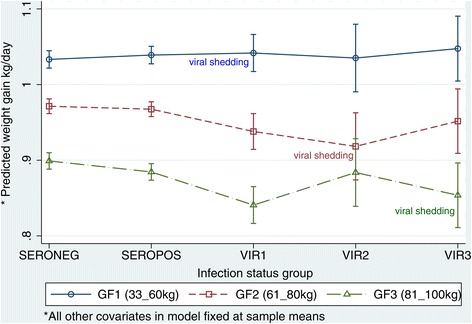
**Marginal plots for average daily growth (ADG) and adverse effects of influenza A(H1N1)pdm09 virus infection.** Based on the model presented in Table 2, the mean ADG was predicted for each of the five infection status groups and the three growth phases (GF), while all other covariates of birthdate, feed intake and breed were fixed at the sample means. The effect of the virus infection is marked by comparing the four infected groups with the reference seronegative group on the same growth phase denoted by the line joining the groups. Comparisons between groups were made for each growth phase since ADG vary with age. As depicted in the graph, the younger pigs would hypothetically have a higher ADG because they have a better FCE (see Figure 2 and Table 3) if feed intake is fixed at the same level. The gradient of the lines joining the means of each group and the confidence intervals indicate whether there were differences between the groups. Abbreviations: INFGP = Infection status group; SEROPOS = seropositive pigs, SERONEG = seronegative pigs, VIR1 = PCR-positive pigs at bodyweight between 33 kg and 60 kg (GF1); VIR2 = PCR-positive pigs at bodyweight between 61 kg and 80 kg (GF2); VIR3 = PCR-positive pigs at bodyweight between 81 kg and 100 kg (GF3).

**Figure 3:**
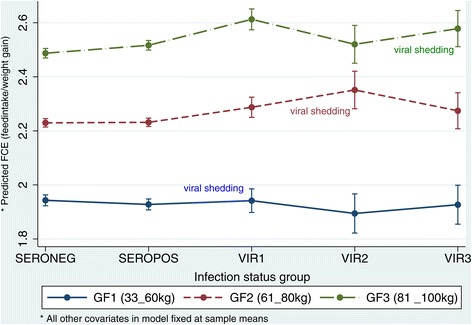
**Marginal plots for feed conversion efficiency (FCE) and adverse effects of influenza A(H1N1)pdm09 virus infection.** Based on the model presented in Table 3, the mean FCE was predicted for each of the five infection status groups and the three growth phases (GF) while all the other covariates of birthdate, feed intake and breed were fixed at the sample means. The effect of the virus infection is marked by comparing the four infected groups with the reference seronegative group on the same growth phase as denoted by the line joining the groups. Comparisons were made for each growth phase since feed efficiency decreases with age. The gradient of the lines joining the means of each group and the confidence intervals indicate whether there were differences between the groups. Abbreviations: INFGP = Infection status group; SEROPOS = seropositive pigs, SERONEG = seronegative pigs, VIR1 = PCR-positive pigs at bodyweight between 33 kg and 60 kg (GF1); VIR2 = PCR-positive pigs at bodyweight between 61 kg and 80 kg (GF2); VIR3 = PCR-positive pigs at bodyweight between 81 kg and 100 kg (GF3).

Tables 2, 3 and 4 show that with SERONEG pigs as the reference, there were statistically significant marginal adverse effects on ADG and FCE in all infected groups (SEROPOS and VIRs 1–3). The marginal plots in Figures 2 and 3 show the predicted means of ADG and FCE while the covariates of BD, ADFI and Br were kept at the sample means. Only GF and INFGP were allowed to vary so that the effect of the virus infection on ADG and INFGP could be studied in the three strata of growth phases. The differences in the predicted FCE and ADG between the five groups of pigs in each of the three growth phases can then be attributed to infection status of the pig.

**Table 2:** **Multilevel regression of average daily growth in pigs infected with influenza A(H1N1)pdm09 virus**

**Average Daily Growth (ADG)**					
**Predictors**	**Coefficients**	**SE**	**P values**	**95% CI**	
**Breed**					
Landrace	**Reference**				
Duroc	−0.028	0.006	<0.001	−0.039	−0.016
**Growth phase**					
GF1 (33_60 kg)	**Reference**				
GF2 (61_80 kg)	−0.062	0.0071	<0.001	−0.08	−0.05
GF3 (81_100 kg)	−0.134	0.0083	<0.001	−0.15	−0.12
**Birthdate**	0.00006	0.000006	<0.001	0.00005	0.00007
**Average daily feed intake**	0.31	0.0053	<0.001	0.30	0.32
Interaction term: INFGP*GF					
SERONEG*GF(1–3)	**Reference**				
SEROPOS*GF1	0.006	0.0064	0.37	−0.01	0.02
SEROPOS*GF2	−0.004	0.0064	0.54	−0.02	0.01
SEROPOS*GF3	−0.015	0.0064	0.02	−0.03	0.00
VIR1*GF1	0.008	0.0127	0.51	−0.02	0.03
VIR1*GF2	−0.033	0.0127	0.01	−0.06	−0.01
VIR1*GF3	−0.058	0.0127	<0.001	−0.08	−0.03
VIR2*GF1	0.002	0.0230	0.94	−0.04	0.05
VIR2*GF2	−0.053	0.0230	0.02	−0.10	−0.01
VIR2*GF3	−0.015	0.0230	0.51	−0.06	0.03
VIR3*GF1	0.014	0.0221	0.52	−0.03	0.06
VIR3*GF2	−0.020	0.0221	0.37	−0.06	0.02
VIR3*GF3	−0.045	0.0221	0.04	−0.09	0.00
**Constant (β**_**0**_ **)**	−0.746	0.1113	<0.001	−0.96	−0.53

*Abbreviations*: *SERONEG* Seronegative pigs; *SEROPOS* Seropositive pigs (not tested for virus), *VIR1* pigs viral positive between bodyweight 33 kg and 60 kg, *VIR2* pigs viral positive between bodyweight 61 kg and 80 kg, *VIR3* pigs viral positive between bodyweight 81 kg and 100 kg.

The model is hierarchical with three levels; observations (n = 5865); pig (n = 1955) and herd (n = 43). *denotes interaction term.

**Table 3:** **Multilevel regression of feed conversion efficiency in pigs infected with influenza A(H1N1)pdm09 virus**

**Feed Conversion Efficiency (FCE)**					
**Predictors**	**Coefficients**	**SE**	**P value**	**95% CI**	
**Breed**					
Landrace	**Reference**				
Duroc	0.0426	0.010	<0.001	0.0230	0.0622
**Growth phase**					
GF1(33_60 kg)	**Reference**				
GF2(61_80 kg)	0.2865	0.011	<0.001	0.26	0.31
GF3(81_100 kg)	0.5442	0.014	<0.001	0.52	0.57
**Birthdate**	-0.0002	0.00001	<0.001	-0.0002	-0.0001
**Average daily feed intake**	0.0238	0.010	0.019	0.004	0.044
Interaction term: INGP*GF					
SERONEG*GF(1-3)	**Reference**				
SEROPOS*GF1	-0.015	0.011	0.159	-0.037	0.006
SEROPOS*GF2	0.002	0.010	0.849	-0.018	0.022
SEROPOS*GF3	0.029	0.010	0.004	0.010	0.049
VIR1*GF1	-0.001	0.023	0.951	-0.046	0.043
VIR1*GF2	0.058	0.020	0.004	0.018	0.097
VIR1*GF3	0.125	0.020	<0.001	0.086	0.165
VIR2*GF1	-0.049	0.037	0.189	-0.121	0.024
VIR2*GF2	0.122	0.036	0.001	0.051	0.192
VIR2*GF3	0.033	0.036	0.361	-0.038	0.103
VIR3*GF1	-0.016	0.037	0.659	-0.089	0.056
VIR3*GF2	0.045	0.035	0.196	-0.023	0.112
VIR3*GF3	0.091	0.035	0.008	0.023	0.159
**Constant (β**_**0**_ **)**	4.6609	0.1934	<0.001	4.282	5.040

*Abbreviations*: *SERONEG* Seronegative pigs, *SEROPOS* Seropositive pigs (not tested for virus), *VIR1* pigs viral positive between bodyweight 33 kg and 60 kg, *VIR2* pigs viral positive between bodyweight 61 kg and 80 kg, *VIR3* pigs viral positive between bodyweight 81 kg and 100 kg. The model is hierarchical with three levels; observations (n = 5865); pig (n = 1955) and herd (n = 43). *denotes interaction term.

For SEROPOS pigs, the negative effect on growth performance was seen during GF3 where FCE was reduced by +0.029 kg feed/kg weight gain. Correspondingly, ADG also decreased by −0.015 kg/day.

For VIR1 pigs, adverse growth performance effects were not seen until GF2 even though these pigs were positive for virus during GF1. During GF2, FCE was reduced by +0.058 kg feed/kg weight gain, which led to a lower ADG by −0.033 kg/day. These negative effects extended into GF3 where FCE was reduced by +0.125 kg feed/kg weight gain and a corresponding lower ADG by −0.058 kg/day.

Removing twenty-two oldes pigs in VIR1 pigs to to leave 101 younger pigs (VIR1a) that were viral positive when they were 50 kg or less did not make any difference to the results we saw in Table 3 in that the delayed adverse effects were seen in GF2 and worsen in GF3 (Table 4).

**Table 4:** **Multilevel regression of feed conversion efficiency in pigs infected with influenza A(H1N1)pdm09 virus**

**Feed Conversion Efficiency (FCE)**					
**Predictors**	**Coefficients**	**SE**	**P values**	**95% CI**	
**Breed**					
Landrace	**Reference**				
Duroc	0.042	0.010	<0.001	0.023	0.062
**Growth phase**					
GF1(33_60 kg)	**Reference**				
GF2(61_80 kg)	0.287	0.011	<0.001	0.264	0.309
GF3(81_100 kg)	0.544	0.014	<0.001	0.517	0.571
**Birthdate**	-0.0002	0.00001	<0.001	0.000	0.000
**Average daily feed intake**	0.024	0.010	0.02	0.004	0.044
**Interaction term: INFGP**					
SERONEG*GF(1-3)	**Reference**				
SEROPOS*GF1	-0.015	0.011	0.1570	-0.037	0.006
SEROPOS*GF2	0.002	0.010	0.8560	-0.018	0.022
SEROPOS*GF3	0.029	0.010	0.0040	0.009	0.049
VIR1a*GF1	-0.008	0.025	0.7510	-0.057	0.041
VIR1a*GF2	0.050	0.022	0.0230	0.007	0.093
VIR1a*GF3	0.114	0.022	0.0000	0.071	0.157
VIR1b*GF1	0.026	0.049	0.5930	-0.070	0.122
VIR1b*GF2	0.092	0.044	0.0390	0.005	0.178
VIR1b*GF3	0.176	0.045	0.0000	0.087	0.265
VIR2*GF1	-0.049	0.037	0.1870	-0.122	0.024
VIR2*GF2	0.121	0.036	0.0010	0.051	0.192
VIR2*GF3	0.033	0.036	0.3640	-0.038	0.103
VIR3*GF1	-0.016	0.037	0.6560	-0.089	0.056
VIR3*GF2	0.045	0.035	0.1970	-0.023	0.112
VIR3*GF3	0.091	0.035	0.0090	0.023	0.159
**Constant (β**_**0**_ **)**	4.658	0.193	<0.001	4.279	5.037

*Abbreviations*: *SERONEG* Seronegative pigs, *SEROPOS* Seropositive pigs (not tested for virus), *VIR1a* pigs viral positive between bodyweight 33 kg and 50 kg, *VIR1b* pigs viral positive between bodyweight 51 kg and 60 kg, *VIR2* pigs viral positive between bodyweight 61 kg and 80 kg, *VIR3* pigs viral positive between bodyweight 81 kg and 100 kg. The model is hierarchical with three levels; observations (n = 5865); pig (n = 1955) and herd (n = 43). *denotes interaction term.

For VIR2 and VIR3 pigs, the negative effects were confined to the same growth phase that they were positive for virus. For VIR2 pigs during GF2, FCE was reduced by +0.122 kg feed/kg weight and ADG was lower by −0.053 kg/day correspondingly. The FCE and ADG of VIR2 pigs returned to the same levels as seronegative pigs during GF3. For VIR3 pigs during GF3, the FCE was reduced by +0.091 kg feed/kg weight gain and ADG was lower by −0.045 kg/day correspondingly.

### Average daily feed intake

Surprisingly we saw in Table 5 that there was no significant decrease in ADFI for the four infected groups (seropositive group and the three virus positive groups) since anorexia was listed as one of the clinical signs in pigs infected with SIVs. It was also interesting to observe that VIR1 pigs had increased feed intake during the post viral shedding period. Their average daily feed intake increased by 71 g/day and 0.104 kg/day during GF2 and GF3 respectively.

**Table 5:** **Multilevel regression of average daily feed intake in pigs infected with influenza A(H1N1)pdm09 virus**

**Average daily feed intake (ADFI)**					
**Predictors**	**Coefficients**	**SE**	**P values**	**95% CI**	
**Breed**					
Landrace	**Reference**				
Duroc	−0.10	0.0157	<0.001	−0.13	−0.07
**Growth Phase**					
GF1(33_60 kg)	**Reference**				
GF2(61_80 kg)	0.71	0.0136	<0.001	0.68	0.74
GF3(81_100 kg)	1.08	0.0136	<0.001	1.05	1.10
**Birthdate**	−0.00002	0.00002	0.257	−0.00005	0.00001
Interaction term: INFGP*GF					
SERONEG*GF(1–3)	**Reference**				
SEROPOS*GF1	−0.003	0.016	0.851	−0.034	0.028
SEROPOS*GF2	0.022	0.016	0.166	−0.009	0.053
SEROPOS*GF3	0.017	0.016	0.278	−0.014	0.049
VIR1*GF1	−0.024	0.032	0.444	−0.086	0.038
VIR1*GF2	0.071	0.032	0.025	0.009	0.133
VIR1*GF3	0.104	0.032	0.001	0.042	0.166
VIR2*GF1	0.077	0.057	0.176	−0.035	0.190
VIR2*GF2	0.032	0.057	0.574	−0.080	0.144
VIR2*GF3	0.070	0.057	0.223	−0.042	0.182
VIR3*GF1	0.059	0.055	0.283	−0.049	0.167
VIR3*GF2	0.077	0.055	0.164	−0.031	0.184
VIR3*GF3	−0.085	0.055	0.122	−0.193	0.023
**Constant (β**_**0**_ **)**	1.897	0.3107	<0.001	1.288	2.506

*Abbreviations*: *SERONEG* Seronegative pigs, *SEROPOS* Seropositive pigs (not tested for virus), *VIR1* pigs viral positive between bodyweight 33 kg and 60 kg, *VIR2* pigs viral positive between bodyweight 61 kg and 80 kg, *VIR3* pigs viral positive between bodyweight 81 kg and 100 kg.

The model is hierarchical with three levels; observations (n = 5865); pig (n = 1955) and herd (n = 43). *denotes interaction term.

### Age of pigs at 100 kg

**Figure 4:**
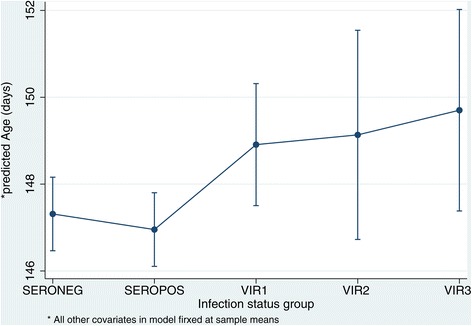
**Marginal plots of mean age at 100 kg and adverse effects of influenza A(H1N1)pdm09 virus infection.** The plots show the predicted mean ages of pigs at 100 kg for the five groups of pigs based on the model presented in Table 6. The gradient of the line joining the mean for each group and the confidence intervals indicate whether there is any difference between the groups due to their infection status. Comparisons were made between the four infected groups and the reference seronegative group. Abbreviations: INFGP = Infection status group; SEROPOS = seropositive pigs, SERONEG = seronegative pigs, VIR1 = PCR-positive pigs at bodyweight between 33 kg and 60 kg (GF1); VIR2 = PCR-positive pigs at bodyweight between 61 kg and 80 kg (GF2); VIR3 = PCR-positive pigs at bodyweight between 81 kg and 100 kg (GF3).

Consequent to the reduced FCE and hence lower ADG, the virus positive pigs required longer time to reach the bodyweight of 100 kg (Figure 4 and Table 6). The VIR1, VIR2 and VIR3 pigs were slower by 1.6 days, 1.8 days and 2.4 days, respectively, in reaching 100 kg bodyweight. Although VIR2 was statistically insignificant (P value = 0.12), it was on similar trajectory as VIR1 and VIR3.

**Table 6:** **Multilevel regression of pig’s age at 100 kg if they are infected with influenza A(H1N1)pdm09 virus**

**Age of pig at 100 kg**					
**Predictors**	**Coefficients**	**SE**	**P values**	**95% CI**	
**Breed**					
Landrace	**Reference**				
Duroc	7.3	0.83	<0.001	5.7	9.0
**Birthdate**	−0.006	0.0005	<0.001	−0.007	−0.005
**Average daily feed intake**	−28.5	0.94	<0.001	−30.3	−26.6
**Infection status group**					
SERONEG	**Reference**				
SEROPOS	−0.4	0.34	0.292	−1.0	0.3
VIR1	1.6	0.67	0.017	0.3	2.9
VIR2	1.8	1.20	0.129	−0.5	4.2
VIR3	2.4	1.15	0.038	0.1	4.6
**Constant (β**_**0**_ **)**	316.8	9.4	<0.001	298.3	335.3

*Abbreviations*: *SERONEG* Seronegative pigs, *SEROPOS* Seropositive pigs (not tested for virus), *VIR1* pigs viral positive between bodyweight 33 kg and 60 kg; VIR2= pigs viral positive between bodyweight 61 kg and 80 kg, *VIR3* pigs viral positive between bodyweight 81 kg and 100 kg.

The model is hierarchical with two levels; pig (n = 1955) and herd (n = 43).

### Overall feed intake

**Figure 5:**
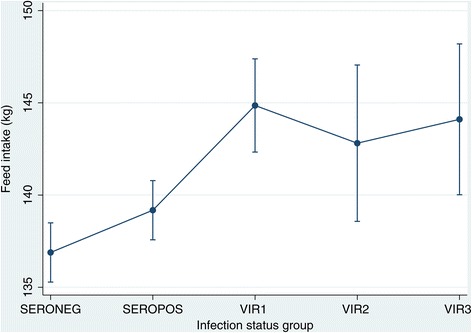
**Marginal plots of overall feed intake and adverse effects of influenza A(H1N1)pdm09 virus infection.** The plots show the predicted mean overall feed intakes for the five groups of pigs based on the model presented in Table 7. The gradient of the line joining the mean for each group and the confidence intervals indicate whether there is any difference between the groups due to their infection status. Comparisons were made between the four infected groups and the reference seronegative group. Abbreviations: INFGP = Infection status group; SEROPOS = seropositive pigs, SERONEG = seronegative pigs, VIR1 = PCR-positive pigs at bodyweight between 33 kg and 60 kg (GF1); VIR2 = PCR-positive pigs at bodyweight between 61 kg and 80 kg (GF2); VIR3 = PCR-positive pigs at bodyweight between 81 kg and 100 kg (GF3).

Consequent to the reduced FCE experienced by the four infected groups, Figure 5 and Table 7 show that all four infected groups needed additional feed to reach 100 kg bodyweight. The SEROPOS, VIR1, VIR2 and VIR3 pigs needed 2.3 kg, 8.0 kg, 5.9 kg and 7.2 kg additional feed, respectively.

**Table 7:** **Multilevel regression for overall feed intake of pig infected with influenza A(H1N1)pdm09 virus**

**Overall feed intake of pig growing from 33 kg to 100 kg (OFI)**					
**Predictors**	**Coefficients**	**SE**	**P values**	**95% CI**	
**Breed**					
Landrace	**Reference**				
Duroc	5.89	1.6	<0.001	2.8	9.0
**Birthdate**	−0.02	0.0009	<0.001	−0.020	−0.016
**Average daily feed intake**	11.74	1.6	<0.001	8.5	14.9
**Infection group**					
SERONEG	**Reference**				
SEROPOS	2.3	0.6	<0.001	1.1	3.5
VIR1	8.0	1.2	<0.001	5.7	10.3
VIR2	5.9	2.1	0.005	1.8	10.0
VIR3	7.2	2.0	<0.001	3.3	11.2
**Constant (β**_**0**_ **)**	445.0	16.5	<0.001	412.7	477.2

*Abbreviations*: *SERONEG* Seronegative pigs, *SEROPOS* Seropositive pigs (not tested for virus), *VIR1* pigs viral positive between bodyweight 33 kg and 60 kg, *VIR2* pigs viral positive between bodyweight 61 kg and 80 kg, *VIR3* pigs viral positive between bodyweight 81 kg and 100 kg.

Growth phase was from 33 kg to 100 kg bodyweight. The model is hierarchical with two levels; pig (n = 1955) and herd (n = 43).

### Other predictors (covariates or control variables) in the models

The predictors birthdate, breed and growth phase were highly significant (P value<0.001) in our regression models for all five outcomes). For birthdate as a predictor, the outcomes of ADG, FCE, ADFI, Age100kg and OFI of pigs improved each year or every 365 days by 0.022 kg bodyweight/day, −0.055 kg feed/kg bodyweight, −0.007 kg feed/day, −2.2 days and −6.2 kg feed respectively.

On breed differences, Landrace pigs grew faster (ADG of +0.028 kg/day), had better efficiency in feed conversion (FCE was improved by −0.043 kg feed/kg bodyweight), reached the targeted weight seven days earlier, and required less feed (−5.9 kg feed) to reach 100 kg bodyweight.

### Clinical observations between April and July 2011

During a clinical outbreak at the station from April 2011 to July 2011, a group 2045 pigs present at the station were monitored closely for clinical signs. These pigs entered the station at different time since the station received 72 new pigs on a weekly basis. Influenza-like illness was observed in 137 pigs, giving a crude morbidity of 7%. Clinical signs observed were transient anorexia or lethargy (45%), respiratory signs (coughing, laboured breathing or nasal discharge, 39%), and pyrexia (above 39°C, 27%). The prevalence of these clinical signs recorded within each of the 16 rooms ranged from 0% to 17%. For group specific morbidity, clinical signs were not detected in VIR1 pigs, while one (3%) out of 34 VIR2 pigs and five (14%) out of 37 VIR3 pigs had clinical signs reported. In the seropositive group, 39 (10%) out of 381 pigs had clinical signs of influenza-like illness during the testing period. No clinical signs were reported in the SERONEG pigs.

## Discussion

The findings in our study support the alternate hypothesis that despite being a largely subclinical disease (<4% morbidity in 194 viral positive pigs), influenza A(H1N1)pdm09 virus infection reduced the pigs’ growth performance in terms of FCE and hence ADG. Consequently the infected pigs needed more time to reach 100 kg and additional feed was also consumed. The negative growth performance effects were most evident in the pigs that were infected at a young age, as shown by the VIR1 pigs. The negative effects of reduced FCE and ADG in VIR1 extended into growth phases two and three. That was beyond the viral shedding period of SIVs of about seven days [7]. We have no explanation why adverse effects in growth performance appeared only during the post viral period and lasted longer in these VIR1 pigs that were infected at bodyweight before 60 kg bodyweight. The duration of negative effects were shorter in the pigs infected at a later age as represented by VIR2 pigs. Negative effects in these pigs were limited to GF2, the same growth phase that they were tested positive for the virus. Twenty two of the 123 VIR1 pigs were in the upper weight range of 50 kg and 60 kg. If these pigs were in the early viral shedding period of 7 days during testing, shedding of virus and presumably the manifestation of adverse effects on growth performance could cross over to the next growth phase (61-80 kg). Misclassication bias could then result in concluding the delayed and extended negative effects of the virus infection on FCE in VIR1 pigs (Table 3). However in our bias analysis (Table 4), the removal of these twenty-two heavier pigs, from VIR1 and leaving 101 younger pigs (VIR1a) that shed virus between 33 kg and 50 kg did not change the result. Just as in the original VIR1 group of 123 pigs, the adverse effects on growth performance of these 101 younger pigs (VIR1a) appeared during the post viral period of growth phase two (61 -80 kg) and deteriorated during growth phase three (81 -100 kg).

Interestingly, none of the infected groups had depressed average feed intake in any of the three growth phases as we expected because anorexia is listed as one of the clinical signs in pigs infected with the classical SIVs. Instead the pigs infected at a young age (VIR1) ate more during growth phases two and three, which were the post viral shedding period for VIR1 pigs. Even though they increased their feed intake, it was insufficient to compensate for the reduced FCE during growth phases 2 and 3 to raise their daily growth high enough to catch up with the seronegative pigs. Consequently the overall feed intake of these VIR1 pigs increased by 8 kg but were still slower by 1.6 days in getting to 100 kg bodyweight. Despite their lower FCE, the increased appetite of VIR1 pigs during the post viral shedding period of GF2 and GF3 was enough to allow them to reach the bodyweight of 100 kg earlier than VIR2 and VIR3 pigs which were infected at a later age.

Landrace and Duroc are the two major breeds in Norway. The proportions of both breeds were almost equal in our study. Even though there were intrinsic breed differences in growth profile with Landrace having a better feed conversion efficiency and higher daily feed intake and hence higher daily growth, our investigation found no differential adverse effects on growth performance caused by this virus infection on the two breeds.

Although the time period of infection for the 874 pigs in the SEROPOS group were unknown, these pigs could have been infected at any time during their growth phase before or after arriving at the boar station. Blood for cELISA tests were collected from these pigs when they were about 100 kg. With the exception of five pigs (67 days for the youngest) in the SEROPOS group, the remaining 869 pigs were older than 12 weeks when they were tested for antibodies. It was therefore unlikely that they harboured detectable maternal antibodies [24-26] at the time of testing. The observation that the SEROPOS pigs had similar results to the 37 virus positive pigs in the VIR3 group in that the adverse effect on FCE and ADG occurred during GF3 points to the possibility that the majority of seropositive pigs were infected at the older age like VIR3 pigs. Although these SEROPOS pigs reached 100 kg bodyweight at the same time as the SERONEG pigs, these SEROPOS pigs had reduced FCE during growth phase three which resulted in these pigs consuming an additional 2 kg feed to reach the bodyweight of 100 kg.

### Bias analysis on misclassification

The cELISA test had a sensitivity and specificity of 93.7% and 99.1% (Manufacterers data sheets). Although these values are considered high for test performance, given the largely lack of clinical picture in this disease for corroboration, there could nevertheless be a small number of SERONEG and SEROPOS pigs that were misclassified and hence biased the adverse effects towards the null. Our quantitative bias analysis using Episens [23] for adverse effects on a dichotomized FCE showed that the odds ratio for a poorer FCE if the pig was SEROPOS versus SERONEG was 1.13. The odds ratio after adjusting for misclassification bias was 1.3, a change of 2 percent. The small bias of 2 percent in our study was towards the null (OR= 1).

The literature [7] states that SIVs in general, cause near if not 100% morbidity when they infect naïve pigs. An experimental study with influenza A(H1N1)pdm09 virus by Brookes et al. in 2010 [9] reported 100% morbidity involving 19 pigs in their experimental study, where 2 pigs were infected by contact. In contrast, we found in our field study that influenza A(H1N1)pdm09 virus infection of Norwegian pigs was largely subclinical, with only six (<4%) in a sample of 194 virus positive pigs reported to have clinical signs. Apart from coughing, clinical signs, like nasal discharge, were so mild that only through closer observation or handling of the pig, for example nasal swabbing, could the signs be detected. The mild clinical signs and low morbidity recorded during the observation period were similar to the morbidity experienced by other Norwegian pig farms infected with influenza A(H1N1)pdm09 virus for the first time [15,16]. This shows that despite Norwegian pigs having no cross-protective immunity [27] to other strains of SIVs, the A(H1N1)pdm09 virus experienced in Norwegian pigs appeared to be of low pathogenicity that caused no or only mild clinical signs.

In recording the clinical signs at the pig testing station, possible bias of focusing on pigs with anorexia (shown on the computer records) may have led to underestimating the morbidity of the disease because we found no statistically significant decrease in appetite in the infected pigs in the stipulated three growth phases. A transient drop in appetite for one or two days would be masked by the three growth phases since the intervals in each growth phase were longer than two days and if there were compensatory increases in feed intake following one or two days of depressed feed intake.

### Favourable conditions in our study

Pigs in our study did not have co-infections of other subtypes of influenza A viruses, *M. hyopneumoniae*, PRRSV, Aujesky’s disease virus and porcine respiratory coronavirus, given Norway’s disease free status for these pathogens [12]. Secondly, the daily recordings of feed intake and bodyweight for each pig were computerised without the presence of human interference. This ruled out human bias or error in making measurements to provide accurate calculations of the performance parameters. Thirdly, the repeated measures allowed the study of progressive effects of the virus in pigs infected at different ages and also the duration of the negative effects on growth performance.

### Validity of study design and statistical models

With 34 or more pigs in each of the five groups of pigs, the sample sizes are considered large for multi-level models [28]. The five statistical models were based on maximum likelihood in estimating the predictors that allow for inference to the Norwegian pig population.

It was appropriate to use multi-level analysis because of the hierarchical nature of the data. The data, having 5865 observations nested in the 1955 pigs, which in turn were nested in the 43 herds, were handled to account for the variations between individual pigs and between the various herds including unmeasured confounders like other infections at the herd or individual level. Pigs from the study sample of 43 herds were represented in the reference group of 887 seronegative pigs. Consequently, the effects of virus infection (primary predictor of interest) and the known covariates (predictors we wanted to control) were more accurately estimated. Such hierarchical models solved some of the problems mentioned in a similar study by Straw et al. [18], in that this study was designed to control heterogeneities due to the environment, herd health status, host characteristics and management conditions inherent at the pig and herd level to reduce if not eliminate confounding [29,30]. Furthermore, keeping the pigs at one location in a uniform environment and husbandry eliminated these factors as potential confounders in our model. Our models also proved to have a relatively high explanatory ability on the variance as the achieved adjusted R^2^ were 51% (ADG), 59% (FCE), 51% (Age100 kg), 66% (ADFI) and 20% (OFI), which were proportions of variation that were explained by the predictors in the models. The longitudinal nature of the data for each pig allowed the statistical models to account for changes to FCE, ADFI and hence ADG with respect to the stage in the pig’s growth phase by including growth phase (GF) as a dummy variable in the statistical models thus controlling for confounding due to normal variation of feed conversion efficiency and daily feed intake with stage in growth phase.

Our samples of 1955 pigs included pigs tested at the station over four years from 2009 and 2012. We saw in our models that birthdate was a significant covariate because pigs born later had better growth performance as a result of improvement over time due to genetic selection, improved feed, and management improvement. Pigs belonging to the five infection status groups were disproportionately distributed over these four years since all 194 virus positive pigs were sampled in a single year (2011) while 560 pigs from seronegative and seropositive group were sampled in 2009 and 2010. Despite these disproportions, we were able to account for the marginal effects attributed to improvement over time by including birthdate as a covariate in our multi-level regression models. This allowed us to increase the study sample and hence the power of our study.

As normal occurrence and also seen in our study, the feed intake increases and FCE declines as a pig grows. Despite the reduced FCE in an older pig, its ADG is still higher because it consumes a higher amount of feed than the younger pig. Hypothetically, if older pigs ate the same amount of feed as the younger pigs, their ADG would be lower because of a reduced FCE as depicted by the coefficients of GF for outcome ADG and FCE in Tables 2, 3 and 4. This is also depicted in our marginal plots at Figures 2 and 3. This again underlines the importance of having growth phase as a dummy variable in our models to ensure comparisons of our outcomes between the 5 infection-status groups were valid because comparisons between groups were made in the same growth phase.

The coefficients of covariates breed, birthdate, growth phase and average daily feed intake in all five models were useful for the validation of our statistical models by comparing their values with other sources of pig performance data. An improbable coefficient would have raised a red flag on the models.

All five outcomes in our models were correlated. The calculation of feed conversion efficiency was based on average daily feed intake and average daily growth recordings. They in turn determined the remaining 2 outcomes on overall growth performance, which were age of pig at 100 kg bodyweight and overall feed intake. These latter 2 outcomes on overall growth performance are especially useful in evaluating economic consequences of the infection for farmers. Cost of extra feed and a delay in getting the pig to the market will lead to higher overheads (feed and veterinary costs) and lower income for the farmer since fewer pigs are sold in the fixed time period. We found that pigs infected when they were young (33 kg - 60 kg) required an additional 8 kg feed and were 1.6 days slower in reaching 100 kg bodyweight. Farmers can estimate the added operational costs if they know that their pigs were infected at a young or older age.

In other parts of the world, SIVs seldom act alone, but with concurrent infections to cause porcine respiratory disease complex where SIVs are the most common primary pathogens [1,2,7,31,32]. Hypothetically, the severity of influenza A(H1N1)pdm09 virus infection in terms of growth performance would be aggravated by concurrent infection of these other respiratory pathogens [7]. On the other hand, these pigs could also be protected from influenza A(H1N1)pdm09 virus infection because of presence of protective immunity against other strains of SIVs [27].

## Conclusions

Our study shows that influenza A(H1N1)pdm09 virus infection in Norwegian pigs differs from the classical swine influenza experienced in other parts of the world because of the low morbidity and mild clinical signs. Although largely subclinical, the infection in Norwegian pigs did experience adverse effects on growth performance primarily because of reduced FCE. This is an important consideration for farmers because it directly influences the profitability of the pig production in terms higher overheads in terms of feed costs and additional time needed for infected pigs to reach market weight leading to lower income. The adverse effects were more severe and lasted longer in pigs infected at a younger age.

## Abbreviations

AIC: Akaike Information Criterion
ADFI: Average daily feed intake
ADG: Average daily weight gain
Br: Breed
FCE: Feed conversion efficiency
FIRE: Feed intake recording equipment
GF: Growth phase
INFGP: Infection status group
MJ: Megajoules
OFI: Overall feed intake from 33 kg to 100 kg bodyweight
SERONEG: Negative for antibodies against the virus
SEROPOS: Positive for antibodies against the virus
SIV: Swine influenza virus
VIR1: Pigs positive for the virus between 33 kg and 60 kg bodyweight
VIR2: Pigs positive for the virus between 61 kg and 80 kg bodyweight
VIR3: Pigs positive for the virus between 81 kg and 100 kg bodyweight

## References

[1] Van Alstine WG: **Respiratory System.** In *Disease of Swine.* 10th edition. Edited by Zimmerman J, Karriker L, Ramirez A, Schwartz KJ. Wiley-Blackwell; 2012.

[2] Loeffen WLA, Heinen PP, Bianchi ATJ, Hunneman WA, Verheijden JHM (2003). Effect of maternally derived antibodies on the clinical signs and immune response in pigs after primary and secondary infection with an influenza H1N1 virus. Vet Immunol Immunopathol.

[3] Khatri M, Goyal SM, Saif YM (2012). Oct4+ stem/progenitor swine lung epithelial cells are targets for influenza virus replication. J Virol.

[4] Crisci E, Mussá T, Fraile L, Montoya M (2013). Review: influenza virus in pigs. Mol Immunol.

[5] Torremorell M, Allerson M, Corzo C, Diaz A, Gramer M (2012). Transmission of influenza a virus in pigs. Transbound Emerg Dis.

[6] Garten RJ, Davis CT, Russell CA, Shu B, Lindstrom S, Balish A, Sessions WM, Xu XY, Skepner E, Deyde V, Okomo-Adhiambo M, Gubareva L, Barnes J, Smith CB, Emery SL, Hillman MJ, Rivailler P, Smagala J, de Graaf M, Burke DF, Fouchier RA, Pappas C, Alpuche-Aranda CM, López-Gatell H, Olivera H, López I, Myers CA, Faix D, Blair PJ, Yu C, Keene KM (2009). Antigenic and genetic characteristics of swine-origin 2009 a(H1N1) influenza viruses circulating in humans. Science.

[7] Van Reeth K, Brown IH, Olsen CW, Zimmerman J, Karriker LA, Ramirez A, Schwartz K, Stevenson G (2012). Influenza Virus. Diseases of Swine.

[8] Brookes SM, Brown IH (2011). A/H1N1/pdm09 virus: dynamics of infection in pigs and people. Vet Rec.

[9] Brookes SM, Núñez A, Choudhury B, Matrosovich M, Essen SC, Clifford D, Slomka MJ, Kuntz-Simon G, Garcon F, Nash B, Hanna A, Heegaard PMH, Quéguiner S, Chiapponi C, Bublot M, Garcia JM, Gardner R, Foni E, Loeffen W, Larsen L, Van Reeth K, Banks J, Irvine RM, Brown IH (2010). Replication, pathogenesis and transmission of pandemic (H1N1) 2009 virus in Non-immune pigs. PLoS One.

[10] Lange E, Kalthoff D, Blohm U, Teifke JP, Breithaupt A, Maresch C, Starick E, Fereidouni S, Hoffmann B, Mettenleiter TC, Mettenleiter TC, Beer M, Vahlenkamp TW (2009). Pathogenesis and transmission of the novel swine-origin influenza virus A/H1N1 after experimental infection of pigs. J Gen Virol.

[11] Thacker EL, Thacker BJ, Janke BH (2001). Interaction between Mycoplasma hyopneumoniae and swine influenza virus. J Clin Microbiol.

[12] Lium B, Er C, Zerihun A, Sviland S, Hellberg H (2014). The Surveillance and Control Programme for Specific Virus Infections in Swine Herd in Norway 2013. Surveillance and Control Programmes for Terrestrial and Aquatic Animals in Norway Annual Report 2012.

[13] Grontvedt CA, Er C, Gjerset B, Hauge AG, Brun E, Jorgensen A, Lium B, Framstad T (2013). Influenza A(H1N1)pdm09 virus infection in Norwegian swine herds 2009/10: the risk of human to swine transmission. Prev Vet Med.

[14] Hofshagen M, Gjerset B, Er C, Tarpai A, Brun E, Dannevig B, Bruheim T, Fostad IG, Iversen B, Hungnes O, Lium B (2009). Pandemic influenza A(H1N1)v: human to pig transmission in Norway?. Euro Surveill.

[15] Gjerset B, Er C, Lotvedt S, Jorgensen A, Hungnes O, Lium B, Germundsson A (2011). Experiences after twenty months with pandemic influenza a (H1N1) 2009 infection in the naive Norwegian Pig population. Influenza Res Treat.

[16] Grontvedt CA, Er C, Gjerset B, Germundsson A, Framstad T, Brun E, Jorgensen A, Lium B (2011). Clinical impact of infection with pandemic influenza (H1N1) 2009 virus in naive nucleus and multiplier Pig herds in Norway. Influenza Res Treat.

[17] Kitikoon P, Nilubol D, Erickson BJ, Janke BH, Hoover TC, Sornsen SA, Thacker EL (2006). The immune response and maternal antibody interference to a heterologous H1N1 swine influenza virus infection following vaccination. Vet Immunol Immunopathol.

[18] Straw BE, Shin SJ, Yeager AE (1990). Effect of pneumonia on growth-rate and feed-efficiency of minimal disease pigs exposed to actinobacillus-pleuropneumoniae and mycoplasma-hyopneumoniae. Prev Vet Med.

[19] Wetten M, Odegard J, Vangen O, Meuwissen TH (2012). Simultaneous estimation of daily weight and feed intake curves for growing pigs by random regression. Animal.

[20] Schnyder U, Hofer A, Labroue F, Kunzi N (2001). Genetic parameters of a random regression model for daily feed intake of performance tested French landrace and large white growing pigs. Genet Sel Evol.

[21] USCGf: **What Happens if you Omit the Main Effect in a Regression Model With an Interaction?** In 2012. http://www.ats.ucla.edu/stat/stata/faq/no_main_effect.htm.

[22] Burnham KP, Anderson DR (2002). Model Selection and Multi-Model Inference: A Practical Information - Theoretic Apprach.

[23] Orsini N, Bellocco R, Greenland S (2006). EPISENS: Stata Module for Basic Sensitivity Analysis of Epidemiological Results. Statistical Software Components.

[24] Wellenberg GJ, Bouwkamp FT, Wolf PJ, Swart WAJM, Mombarg MJ, de Gee ALW (2010). A study on the severity and relevance of porcine circovirus type 2 infections in Dutch fattening pigs with respiratory diseases. Vet Microbiol.

[25] Wesley RD, Lager KM (2006). Overcoming maternal antibody interference by vaccination with human adenovirus 5 recombinant viruses expressing the hemagglutinin and the nucleoprotein of swine influenza virus. Vet Microbiol.

[26] Markowska-Daniel I, Pomorska-Mól M, Pejsak Z (2011). The influence of age and maternal antibodies on the postvaccinal response against swine influenza viruses in pigs. Vet Immunol Immunopathol.

[27] Busquets N, Segalés J, Córdoba L, Mussá T, Crisci E, Martín-Valls GE, Simon-Grifé M, Pérez-Simó M, Pérez-Maíllo M, Núñez JI, Abad FX, Fraile L, Pina S, Majó N, Bensaid A, Domingo M, Montoya M (2010). Experimental infection with H1N1 European swine influenza virus protects pigs from an infection with the 2009 pandemic H1N1 human influenza virus. Vet Res.

[28] Snijders TAB, Bosker RJ (2012). Multilevel Analysis: An Introduction to Basic and Advanced Multilevel Modeling.

[29] Dohoo IR, Martin W, Stryhn H (2010). Veterinary Epidemiologic Research.

[30] Diggle P, Liang K, Zeger S: **Analysis of Longitudinal Data.** In Edited by Atkinson AC, Copas JB, Pierce DA, Schervish MJ, Titterington DM. Oxford: Clarendon Press; 1996:64–68.

[31] Loving CL, Brockmeier SL, Vincent AL, Palmer MV, Sacco RE, Nicholson TL (2010). Influenza virus coinfection with Bordetella bronchiseptica enhances bacterial colonization and host responses exacerbating pulmonary lesions. Microb Pathog.

[32] Done SH (1991). Environmental factors affecting the severity of pneumonia in pigs. Vet Rec.

